# Mulberroside F from In Vitro Culture of Mulberry and the Potential Use of the Root Extracts in Cosmeceutical Applications

**DOI:** 10.3390/plants12010146

**Published:** 2022-12-28

**Authors:** Jiralapat Thamrongwatwongsa, Nattaya Pattarapipatkul, Titiradsadakorn Jaithon, Ananya Jindaruk, Atchara Paemanee, Nattanan Panjaworayan T-Thienprasert, Wannarat Pornsiriwong Phonphoem

**Affiliations:** 1 Department of Biochemistry, Faculty of Science, Kasetsart University, Bangkok 10900, Thailand; 2 Metabolomics Research Team, National Omics Center, National Science and Technology Development Agency, Pathum Thani 12120, Thailand

**Keywords:** anti-tyrosinase, callus, cytotoxicity, mulberroside F, *Morus* spp.

## Abstract

Mulberry (*Morus* spp.) is primarily used in sericulture, and its uses also extend to the food, pharmaceutical, and cosmetic industries. Mulberry extracts are rich in many bioactive compounds that exhibit a wide range of biological properties. Mulberroside F (Moracin M-6, 3′-di-O-β-D-glucopyranoside), one of the bioactive compounds found in mulberry, has previously been reported as a whitening agent by inhibiting melanin synthesis and exhibiting antioxidant effects. However, there is still limited information on the presence of this compound in plants cultured in vitro. In this study, the mulberroside F content, biochemical, and cytotoxic properties of the extracts from mulberry cultured in vitro were determined. The results revealed that both root and callus were found to be a potential source of mulberroside F. Furthermore, the mulberroside F content was positively correlated with the inhibitory effects on tyrosinase activity. Cell viability assay also revealed that crude extract of the mulberry root has no cytotoxicity in both human keratinocyte cell line (HaCaT) and Vero cells. Taken together, mulberry tissue culture represents a possible alternative and continuous production of mulberroside F, which could be further utilized in cosmeceutical applications.

## 1. Introduction

Plant-derived ingredients have gained considerable interest, due to their safeness and value, which could be used in several types of industries, such as food and pharmaceuticals. Their uses also extend to cosmeceuticals, which represent cometic products with pharmaceutical actions [1,2,3]. Many cosmeceutical agents with photoprotection activities have been developed for the prevention of skin-aging caused by UV radiation. Photoprotection has been considered an essential mechanism for reducing the effects caused by UV radiation. Previous studies demonstrated that plant-derived ingredients have several benefits, such as UV absorption capacity, high antioxidants, and fewer side effects compared to synthetic sunscreen agents [4]. Some examples are quercetin [5], apigenin [1], arbutin [6], and curcumin [7].

Mulberry (*Morus* spp.) is a tropical plant growing naturally and under cultivation in many countries, such as China, Japan, India, and Thailand. It is well documented in some traditional and folk Chinese medicine. This plant can be classified into two groups according to its uses: mulberries that are grown for feeding the silkworm (*Bombyx mori*) for silk production, and mulberries for human consumption. Mulberry fruits are rich in carbohydrates, vitamins, dietary fibers, and many bioactive compounds [8], whereas the leaves have been considered an excellent source of antioxidants [9]. Furthermore, other parts of the plant, such as stem bark and root, are reported to have many biological activities [10,11]. Several bioactive compounds have been identified in this plant. Due to their potent biological activities, these compounds have become of interest to the pharmaceutical and cosmetic industries [12].

Ultraviolet (UV) radiation may cause several types of skin damage, including sunburn, photoaging, and skin cancer, in the worst-case scenario [13]. Natural substances, plants for instance, have the potential to be used in sunscreen products as a photoprotective agent. A previous study demonstrated that mulberroside F (Moracin M-6, 3′-di-O-β-D-glucopyranoside), a secondary metabolite that belongs to the class of organic compounds known as 2-arylbenzofuran flavonoids, has been found in leaves, roots, and stem barks [14,15]. This compound can inhibit melanogenesis by acting as an anti-tyrosinase, the enzyme involved in the melanin production process in the skin and the antioxidant mechanism [14,15,16]. This data suggested that mulberroside F has the potential to be developed for cosmeceutical uses. Tyrosinase (EC 1.14.18.1), also known as phenol oxidase, is the immediate enzyme responsible for the enzymatic browning and melanogenesis represented in animals, plants, and microorganisms [17]. This enzyme catalyzes two major reactions: the hydroxylation of monophenols to o-diphenols (monophenolase or cresolase activity) and the oxidation of o-diphenols to o-quinones (diphenolase or catecholase activity).

In addition, various biological and pharmacological activities of extracts from different parts of mulberry plants have been reported. For example, acetonic extract of *Morus nigra* L. root barks demonstrated antinociceptive activity in mice [18], whereas 70% ethanol extract of *Morus alba* fruits indicated a protective effect against cisplatin-induced kidney cell damage in pig cell line [19]. However, compared to field cultivation, only a few studies have reported the biological activity of in vitro mulberry cultures.

Plant tissue culture is now a reliable and affordable method for producing bioactive compounds with industrial significance [20,21,22]. This technique has been utilized for in vitro plant cultivation under a controllable environment, allowing for the production of multiple plants in a short period [23]. Several bioactive compounds have been produced using plant tissue culture systems. These include ginsenoside from *Panax ginseng* [24,25], paclitaxel from *Taxus baccata* [26], and resveratrol from *Arachis hypogaea* and *Vitis vinifera* [27]. In mulberry, a tissue culture system has been established to produce mulberroside A [28], 1-deoxynojirimycin (DNJ) [29], and flavonoids [30]. Nevertheless, the potential of mulberry tissue for mulberroside F production has yet to be reported.

Therefore, this research aimed to determine the content of mulberroside F in different parts of the mulberry and investigate biochemical properties and cytotoxicity of root and callus extracts on human tissue culture cells.

## 2. Results

### 2.1. Investigation of Mulberroside F Content in Mulberry

**Figure 1:**
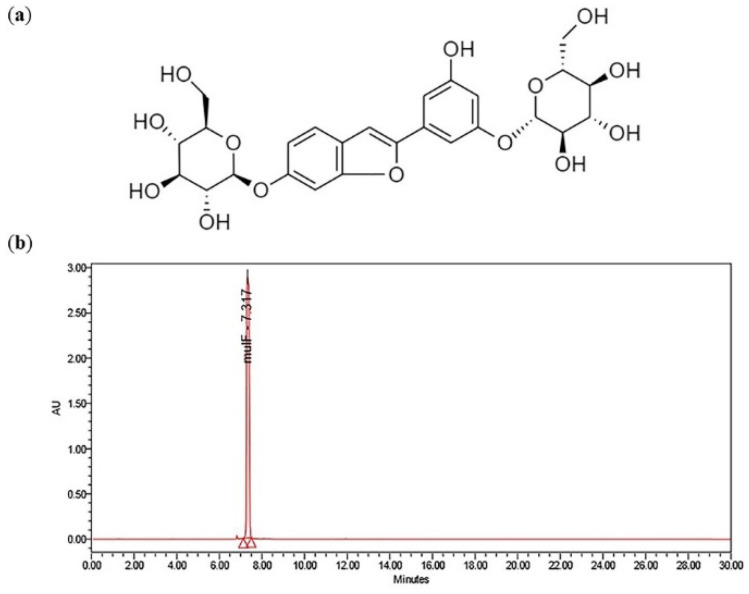
Mulberroside F (moracin M-6, 3′-di-O-beta-D-glucopyranoside) from *Morus* species. (**a**) Chemical structure; (**b**) HPLC chromatogram of mulberroside F standard.

Mulberroside F is a diglycosidic phenolic compound of moracin M and benzofuran derivative. The mulberroside F content was preliminary studied in the soil-grown mulberry. Leaves and roots were harvested, freeze-dried, and subsequently used for extraction, before being subjected to HPLC analysis. The mulberroside F structure is shown in Figure 1a. The working calibration curve was generated from 0–100 µg mL^−1^ (R^2^ = 0.998). The sample mulberroside F was identified by comparing with the retention time (7.3 min) in a standard sample (Figure 1b). It was found that the mulberroside F contents in the leaf and root of soil-grown plants were 30.85 ± 5.98 µg g^−1^ and 121.52 ± 6.59 µg g^−1^, respectively.

### 2.2. Mulberroside F Content in Different Parts of Mulberry Cultured In Vitro

**Figure 2:**
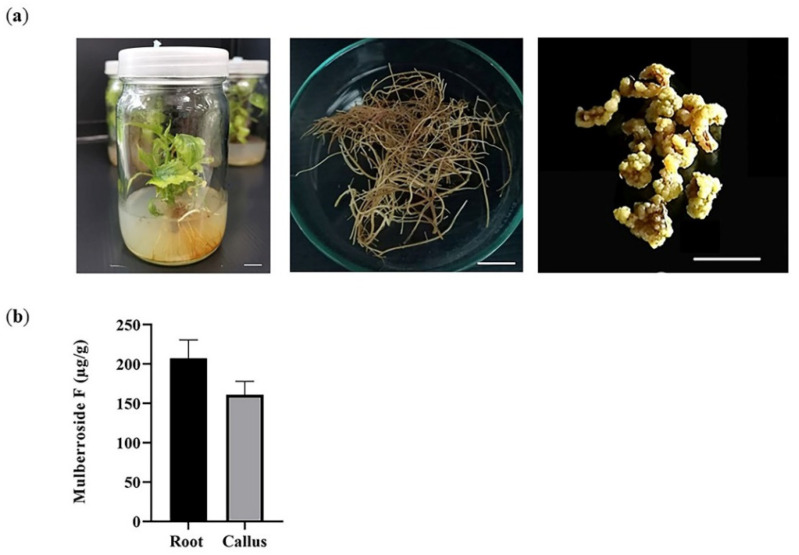
Quantification of mulberroside F in mulberry cultured in vitro. (**a**) Plant material used in this study. 6-week-old mulberry (left), root tissue (middle), and callus (right); (**b**) HPLC analysis of mulberroside F. Error bars were from 3–5 replicates. Scale bar: 1 cm.

Plant tissue culture has become a potential source for valuable bioactive compound production [21]. Thus, the amount of mulberroside F was investigated in mulberry plants cultured in vitro. Based on the data of soil-grown plants, root tissue was selected and compared with the callus (Figure 2a). The result showed that mulberroside F was found in both root (207.35 ± 23.42 µg g^−1^) and callus (160.97 ± 17.08 µg g^−1^) (Figure 2b). The root extracts contained a slightly higher mulberroside F than the callus; however, this was not statistically significant.

### 2.3. Evaluation of Anti-Tyrosinase Activity

**Figure 3:**
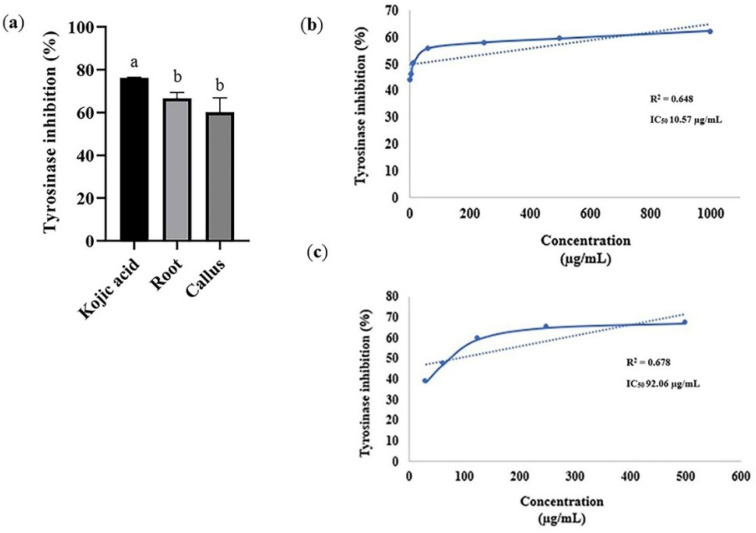
The tyrosinase inhibitory effects of the extracts from root and callus of the mulberry. (**a**) Tyrosinase inhibitory activity of root and callus extracts for the catalysis of L-DOPA. Kojic acid was included as a positive control. Different lowercase letters indicate significant differences between each part analyzed (*p* < 0.05). Error bars represent the standard error of the mean of 3 replicates; (**b**) IC_50_ curve of mulberry root extract; (**c**) IC_50_ curve of callus extract. The values were calculated by nonlinear regression using GraphPad Prism 8.0.1.

In this study, the tyrosinase inhibitory activity of root and callus extracts was investigated using L-3,4-dihydroxyphenylalanine (L-DOPA) as a substrate. At a concentration of 5 mg mL^−1^, the tyrosinase inhibition activities by root and callus were 66.69% and 60.12%, respectively (Figure 3a). Moreover, the IC_50_ values of root and callus extracts were determined, and the values were 10.57 and 92.06 μg mL^−1^, respectively (Figure 3b,c). This result revealed that root and callus extracts possess anti-tyrosinase activity and mulberroside F is one of the bioactive constituents that could contribute to this inhibition.

### 2.4. UV Absorption Capacity

**Figure 4:**
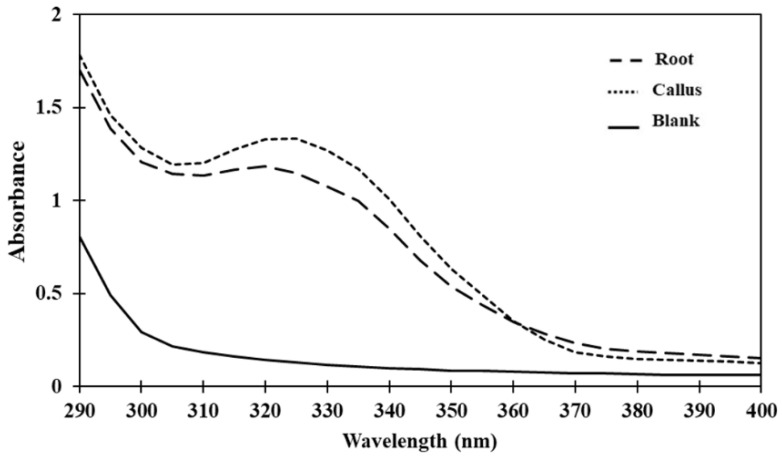
Ultraviolet (UV) absorption spectra of root and callus extracts.

To evaluate the photoprotective activity, the UV absorption of mulberry extracts was determined between the range of 290–400 nm, which cover UVA (320–400 nm) and UVB (290–320 nm) regions, with intervals of 5 nm. As shown in Figure 4, the result suggested that root and callus extracts contain compounds responsible for UV absorption.

### 2.5. Assessment of Cytotoxicity Effects

**Figure 5:**
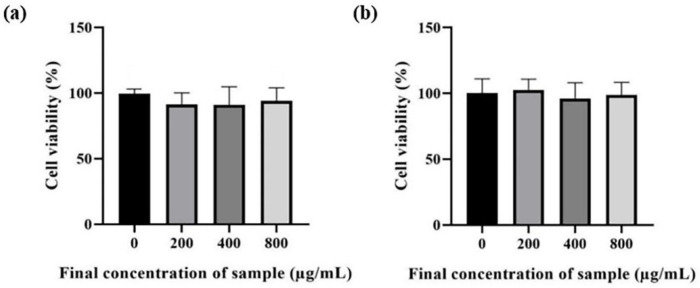
The cytotoxic effects of mulberry root extract on tissue culture cells. (**a**) HaCaT (**b**) Vero. The results are the means of two independent experiments, and each data point was obtained in triplicate. Error bars represent the standard error of the mean.

Next, the cytotoxicity effects of root crude extract of *Morus* spp. were investigated using MTT assay in Vero and HaCaT cells. Vero is an African green monkey kidney cell that is widely used to validate toxicity of toxin [31,32] or cytotoxic effects of drugs [33] and natural products in mammalian cells [34,35]. HaCaT, on the other hand, is a type of immortalized human keratinocyte that has been frequently applied to examine the homeostasis of the epidermis [36,37]. As a result, crude extract of mulberry root showed no cytotoxicity in both HaCaT (Figure 5a) and Vero cells (Figure 5b).

## 3. Discussion

Plant tissue culture systems provide rapid and intensive scale production of phytochemicals for pharmaceutical and cosmeceutical applications [21,38]. In contrast to the conventionally cultivated mulberry, this system can produce uniform and disease-free plants with less variation in the content of bioactive compounds [39].

Using alternative sources such as callus and root cultures has become a new approach for secondary metabolite production. Root has been considered a good source of bioactive compounds, capable of scaling up the production through adventitious root or hairy root culture systems [40,41]. Callus culture is another choice for the enhanced production of secondary metabolites in the pharmaceuticals, cosmetic, and food industries. Several bioactive phytochemicals produced from callus cultures of medicinal plants can be used to treat various diseases, such as cancer, infectious diseases, and cardiovascular diseases [42]. Its advantage also includes the ability to be converted to single-cell suspension culture growing in shake flasks or bioreactors to enhance yield production [43].

Mulberry has long been considered a valuable resource for various food, beverage, cosmetic, and pharmaceutical industries, due to the presence of many phytochemicals with biological functions [11]. Previous studies demonstrated that root barks of mulberry are rich in mulberroside A, kuwanon G, and morusin [44]. Here, the mulberroside F was investigated in different parts of mulberry plants. Previously, mulberroside F was isolated from mulberry leaves. The researchers also reported that this compound could inhibit the activity of tyrosinase, which converts dopa to dopachrome in the biosynthetic activity of melanin [13]. However, there is still a lack of studies regarding the mulberroside F. In this study, the presence of mulberroside F in the roots of mulberry cultured in vitro was reported for the first time. Further, enhancement strategies such as cell suspension culture, hairy root culture, precursor feeding culture, or elicitors [25,45] could be utilized for the production of mulberroside F towards industrial needs.

The primary pigment responsible for the different pigmentations found in animal and human skin, hair, and eyes is melanin, produced by melanocytes via the melanogenesis pathway [46]. Tyrosinase is a rate-limiting, copper-containing enzyme that controls the production of melanin in the human body, which can lead to a variety of skin disorders when overproduced [6]. Thus, the compounds that inhibit melanin synthesis are expected to have cosmetic applications such as whitening agents [47]. Due to skin-whitening properties, the use of tyrosinase inhibitors is becoming increasingly important. Apart from kojic acid, which is commonly known as an inhibitor of tyrosinase, several bioactive compounds from different plant species, including quercetin, chalcones, arbutin, and stilbenes, have also been reported [6,48,49,50]. A previous study showed that the oxyresveratrol, oxyresveratrol-3-*O*-glucoside, and mulberroside A found in mulberry roots could suppress the expression of tyrosinase-related protein-1, and microphthalmia-associated transcription factor (MITF), which involves melanocyte survival and differentiation [51]. The present study further demonstrated that the extracts of root and callus derived from the in vitro culture of mulberry possess anti-tyrosinase activity, and that mulberroside F is one of the bioactive constituents that could contribute to this effect [14]. Additionally, the UV absorption property was also observed, suggesting that the root and callus extracts have a high potential to be used as natural skin care agents. Nevertheless, further study is required to gain insight into the role of mulberroside F on melanogenesis inhibitory mechanism.

Various biological properties of different mulberry extracts have been investigated and reported including medicinal potentials such as anti-cancer activity [52], anti-bacterial activity, and antioxidant activity [53]. Importantly, their toxicity needs to be assessed concurrently for a safe application. According to Li and coworkers (2018), *M. alba* leaf extract had no effect on mortality, hematology, and biochemical parameters in the exposed mice [54]. Moreover, mulberry plant extracts from the leaves and fruits had a protective effect against lead-induced damage to the tissues in rat testes [55]. Additionally, this study showed that culture mulberry root extract was not cytotoxic to Vero and HaCaT cells, but it significantly inhibited tyrosinase activity. Taken together, mulberry extracts include active compounds that may be used in cosmeceutical products.

## 4. Materials and Methods

### 4.1. Chemicals and Reagents

Mulberroside F standard was purchased from Chengdu Biopurify Phytochemicals, China. For anti-tyrosinase assays, mushroom tyrosinase (Sigma-Aldrich, St. Louis, MO, USA), kojic acid (Thermo Fisher Scientific, Waltham, MA, USA), and L-DOPA (Acros organics, Fair Lawn, NJ, USA) were used.

### 4.2. Plant Materials

Mulberry cv. Kamphaeng Saen-MB-42-1 was obtained from the Department of Biochemistry, Faculty of Science, Kasetsart University, Thailand (N 14.015619 E 99.958970), in April 2020. Shoot tips of soil-grown mulberry were excised and used to initiate in vitro culture.

### 4.3. Establishment of Mulberry Tissue Culture

Shoot tips were soaked in 70% ethanol for 1 min and sterilized with 0.1% mercury (II) chloride (HgCl_2_) for 10 min. Then, the explants were washed with distilled water and transferred into Murashige and Skoog (MS) medium (PhytoTechnology, Lenexa, KS, USA), containing 30 g/L of sucrose and 8 g/L of agar powder. The pH of the MS media was adjusted to 5.6–5.8 using 1 M sodium hydroxide (NaOH) (Fisher Chemical, Geel, Belgium) before autoclaving. All plant tissue samples were initially grown in a tissue culture room at 25 ± 1 °C under white fluorescent light (light/dark period of 16/8 h and ~75% RH) for 4–6 weeks.

For callus initiation, the 4-week-old roots from in vitro mulberry plants were excised and placed on solid MS media, supplemented with 0.5 mg/L 2,4-D and 0.5 mg/L BAP. Samples were kept in the dark and sub-cultured every 4 weeks.

### 4.4. Extraction and Quantification of Mulberroside F

Samples of leaves, roots, and callus of mulberry were collected and freeze-dried for 2 days. After that, the samples were powdered by tissue lyser (Retsch GmbH, Haan, Germany). The powdered samples (100 mg) were mixed well in 1 mL of 60 % ethyl alcohol by vortex for 5 min, sonicated in cold water for 20 min, and then incubated for an hour at room temperature. After centrifugation at 13,000 rpm, 4 °C for 10 min, the upper phase was collected to a new tube and stored at −20 °C for further experiments. For anti-tyrosinase activity, UV absorption capacity, and cytotoxicity assays, the extracts were evaporated into dryness with the use of a vacuum centrifuge (Eppendorf, Hamburg, Germany) to obtain the crude extract at a yield of 26%. The extract was resuspended in water prior to use.

HPLC analysis of mulberroside F in leaf, root, and callus was performed using a Waters Alliance Separations Module (Waters, Milford, MA, USA). Mulberroside F were separated on a 4.6 mm x 250 mm, 5 μm particle size, C18 column (Thermo Scientific, Waltham, MA, USA). Solvent A was ultrapure water and solvent B was 100% acetonitrile. The column temperature was maintained at 35 °C with 20 μL of sample. Flow rate was set as 1.0 mL min^−1^ and detected at 320 nm. Samples were filtered before HPLC injection by using 0.2 μm syringe filter. The concentrations of mulberroside F in each part of the mulberry plants were quantified using Empower^®^ 3 software and the results were expressed as μg g^−1^ DW.

### 4.5. Evaluation of Anti-Tyrosinase Activity

Tyrosinase inhibition assays were performed with L-DOPA as substrate. First, the reaction combination of 160 μL of assay mixer containing 120 μL of phosphate buffer (20 mM, pH 6.8), 20 μL of the sample, and 20 μL of mushroom tyrosinase (100 U mL^−1^), was added to each well of the 96-well plate. Then, the microplate was preincubated at 25 °C for 10 min. After that, 20 μL of 2 mM L-DOPA (pH 6.8) was added to each well and further incubated for 20 min. The reaction was observed at 490 nm for dopachrome formation in the reaction mixture. In this experiment, kojic acid was used as a positive control. Each measurement was performed in triplicate (n = 3). The percentage of inhibition was calculated as previously described [56].

### 4.6. Investigation of UV Protective Activity

For the determination of UV-filtering potential, the UV absorption of each extract (1 mg mL^−1^) was measured between 290 and 400 nm, using a Spark^TM^ 10M multimode microplate reader (Tecan, Männedorf, Switzerland).

### 4.7. Cytotoxicity Effects of Normal Cell Cultures

For cell culture and maintenance, normal African green monkey kidney fibroblast cells (Vero; ATCC^®^ CCL-81™) and immortalized human keratinocyte cell line (HaCaT; CLS 300493) were cultured in Dulbecco’s Modified Eagle’s Medium (DMEM; Gibco, Invitrogen, Carlsbad, CA, USA), supplemented with 10 % fetal bovine serum (*v*/*v*), and 1% Antibiotic-antimycotic (*v*/*v*) (Gibco, Invitrogen, CA, USA) at 37 °C in the present of 5% CO_2._

To test the cytotoxicity effects of normal cell cultures, Vero and HaCaT cells were seeded approximately 1.5 × 104 cells per well into 96 well-plates (Falcon^®^ a Corning brand, USA) and incubated for 24 h. All cells were then treated with 0 (distilled water only), 500, 1000, and 1500 μg /mL of crude extract. After 96 h of incubation, the mixing solutions were discarded; then, 4 μg of MTT solution was added and incubated for 3 h. Lastly, MTT solution (Invitrogen, USA) was replaced with 50 μL of dimethyl sulfoxide (Thermo Fisher Scientific, CA, USA) prior to the absorbance measurement at 570 nm by using a Infinite^®^ M Nano microplate reader (Tecan, Männedorf, Switzerland).

### 4.8. Statistical Analysis

Data are presented as the mean ± standard error. The statistical analyses were performed using GraphPad Prism 8.0.1 software. Differences were considered significant at the *p* < 0.05 levels using Tukey’s multiple comparison test.

## 5. Conclusions

Plants have been considered rich sources of biologically active compounds with industry potential. In this study, mulberroside F was identified in the root and callus of mulberry cultured in vitro. The root and callus extracts also have the potential for cosmeceutical applications, due to the anti-tyrosinase activity and the high UV absorption property with no cytotoxic effects. Further studies are necessary for increasing the amount of mulberroside F, for instance, cell suspension culture, precursor feeding, or elicitation strategies.

## Data Availability

Not applicable.
